# Cell Phone Availability and Usage for mHealth and Intervention Delivery to Persons Living With HIV in a Low-Resource Setting: Cross-sectional Study

**DOI:** 10.2196/35631

**Published:** 2022-08-23

**Authors:** Julian Adong, Robin Fatch, Nneka Emenyonu, Winnie Muyindike, Christine Ngabirano, Debbie Cheng, Judith Hahn

**Affiliations:** 1 Faculty of Medicine Mbarara University of Science and Technology Mbarara Uganda; 2 Department of Medicine University of California, San Francisco San Francisco, CA United States; 3 Boston University School of Public Health Boston, MA United States

**Keywords:** cell phone use, phone usage, cell phone, mHealth, HIV, low resource setting, low resource, mobile health, antiretroviral, Uganda, Africa, alcohol, text message, text messaging, cellphone, mobile health, low income, LMIC, TB, tuberculosis, viral infection, infectious disease, sexually transmitted, STD

## Abstract

**Background:**

HIV/AIDS is now a manageable chronic illness owing to effective antiretroviral therapy (ART), which involves routine follow-up care, including regular physical visits to the clinic. In the recent past, and in wake of the COVID-19 pandemic, there has been increased need for virtual care and intervention delivery, a modality known as mobile health (mHealth), which includes cell phone–delivered services for medical and public health practice.

**Objective:**

Here we describe cell phone use and its relationship with alcohol use in a cohort of persons living with HIV and latent tuberculosis (TB).

**Methods:**

We performed a cross-sectional analysis of baseline data from a cohort of persons living with HIV and latent TB in HIV care in southwestern Uganda. We estimated proportions of cell phone and text message use and evaluated their associations with alcohol use—a common modifiable behavior among persons living with HIV. Cell phone use (primary outcome) was defined as owning a cell phone that is turned on at least half of the day. Any alcohol use was defined as any self-reported alcohol use in the prior 3 months or a phosphatidylethanol (an alcohol biomarker) level of ≥8 ng/mL.

**Results:**

A total of 300 participants (median age 40 years; n=146, 48.7% male) were included in the analysis. Most (n=267, 89.0%) participants had access to a phone and of them, 26 (9.7%) shared the phone with someone else. In total, 262/300 (87.3%) of participants owned a cell phone that is turned on at least half of the time; the majority (n=269, 89.7%) rarely or never sent text messages, and over two-thirds (n=200, 66.9%) rarely or never received text messages. Most (n=214, 71.3%) had any alcohol use in the prior 3 months. In adjusted analyses, any alcohol use was not significantly associated with cell phone use (adjusted odds ratio [aOR] 0.48, 95% CI 0.18-1.25; *P*=.13) or sending (aOR 0.82, 95% CI 0.28-2.37; *P*=.71) or receiving (aOR 1.31, 95% CI 0.70-2.47; *P*=.40) text messages.

**Conclusions:**

There is hope that mHealth interventions in this population can be carried out using cell phones owing to their popularity; however, the interventions may need to employ methods that do not rely on the sending and receiving of text messages only.

## Introduction

HIV/AIDS is now a manageable chronic illness owing to the widespread rollout of antiretroviral therapy (ART) [1]. Treatment and prevention of HIV/AIDS involves routine lifelong follow-up care and ongoing interventions to mitigate disease spread and improve treatment outcomes. Follow-up care involves scheduled visits for clinical reviews, medication receipt, laboratory testing, and psychosocial services including counseling and peer support groups. These visits normally involve a physical presence; however, the recent COVID-19 pandemic has made apparent the need for using mobile devices to assist in health and intervention delivery, known as mobile health (mHealth) [2]. mHealth has been studied as both an alternative to and a way to augment physical visits in the chronic care of people living with HIV/AIDS. Recent mHealth interventions for persons living with HIV include cell phone–delivered reminders to promote treatment adherence, motivational messages to encourage clinic attendance and ART adherence, delivery of laboratory tests, and behavior modification messages [3-5]. mHealth interventions may also bridge the service delivery gap during periods of interruptions in valuable HIV/AIDS services including lack of resources for transportation, or more recently, lockdown measures instituted to curb the spread of COVID-19, political unrest, or other epidemic outbreaks including Ebola.

In sub-Saharan Africa where two-thirds of persons living with HIV reside, cell phone access and use has greatly increased over the last decade from an ownership prevalence of 10% in the early 2000s to over 75% in 2019 [6]. In East Africa, cell phone use ranges from approximately 60% in Uganda to over 90% in Kenya [7,8]. Cell phone use gained popularity owing to the widespread coverage quality and ability to reach “everywhere” including resource limited remote areas and the low operational cost. This widespread usage and low cost have been tapped into for mHealth [9]. However, while there has been high enthusiasm for mHealth to improve HIV and other health outcomes, the results of cell phone–delivered interventions among persons living with HIV have been mixed [10,11]. Barriers to the implementation of mHealth interventions may include lack of infrastructure, concerns over privacy and confidentiality, usability issues [12], low level of literacy, low phone ownership, and cost of data to patients [13]. In order to determine the potential for cell phone–delivered interventions for persons living with HIV in low-resource settings, full understanding of cell phone ownership, active use (ie, duration for which a cell phone is kept on and used), and SMS (or texting) use of the targeted intervention population is important as persons living with HIV may differ from the general population in terms of their socioeconomic status and level of literacy [14].

In this analysis, we describe cell phone ownership and use in a cohort of persons living with HIV, who are coinfected with latent tuberculosis (TB) in southwestern Uganda. We also describe the association between alcohol use and cell phone use. Alcohol use and HIV are prevalent in sub-Saharan Africa. Alcohol use affects several HIV treatment outcomes, including ART adherence, viral suppression, and the development of opportunistic infections such as the activation of latent TB, all of which may fuel onward transmission of HIV [15-17]. Alcohol use is, however, a modifiable behavior that could potentially be positively impacted using mHealth interventions [18].

## Methods

### Setting and Population

This was a cross-sectional analysis of baseline data from a study of persons living with HIV who were recruited from a large HIV clinic in southwestern Uganda, entitled the Alcohol Drinkers’ Exposure to Preventative Therapy for Tuberculosis (ADEPTT) study (registered under NCT03302299 in the ClinicalTrials.gov registry). The study was a single-arm prospective trial examining the safety, tolerability, and adherence to a 6-month daily isoniazid (INH) regimen among persons living with HIV who were coinfected with latent TB, who recently consumed alcohol (two-thirds of the sample) or abstained from alcohol consumption (one-third of the sample).

### Study Design

We conducted screening for the ADEPTT study using a multistep process. Eligibility criteria at the initial screening step included being an adult (≥18 years old) living with HIV, being fluent in Runyankole (the local language) or English, having been prescribed ART for at least 6 months, living within 2 hours’ driving distance of or 60 km from the study site, having no plans to relocate from the catchment area, and having no history of active TB or taking TB preventive medications. Alcohol use eligibility additionally included self-reporting current (prior 3 months) alcohol use, or alcohol abstinence (no use in the prior year), with a target of enrolling 200 persons in the former and 100 persons in the latter category. Exclusion criteria are currently taking or having taken nevirapine in the prior 2 weeks, or taking anticonvulsant medications (both contraindications to INH). Further eligibility criteria included alanine transaminase (ALT) and aspartate transaminase (AST) levels being <2× their normal upper limits, being cleared of active TB (for those reporting TB symptoms), and not being pregnant. Finally, participants were screened for evidence of latent TB by research assistants trained in administration and interpretation of the tuberculin skin test (TST), latent TB was defined as a positive TST finding with an induration of ≥5 mm read 48-72 hours after injection with purified protein derivative (PPD). Those with positive TST results were invited to participate in the study. Data included in this analysis are baseline data.

All study participants were reimbursed for transport costs following completion of study procedures at each study visit.

### Ethics Approval

The study was approved by the University of California, San Francisco Institutional Review Board (16-19093), the Mbarara University of Science and Technology Research Ethics Committee (11/10-16), and the Ugandan National Council for Science and Technology (HS2183). Participants provided written consent prior to study participation.

### Measures

Data were collected using an interviewer-administered laptop-based survey using the Computer Assisted Survey Information Collection system. The surveys were conducted in private spaces, for confidentiality reasons, in Runyankole or English, depending on the participants’ preference. Baseline blood draws were conducted for testing for a biomarker of alcohol use—phosphatidylethanol [19]. Phosphatidylethanol is a sensitive and specific marker of prior 3 weeks’ alcohol consumption.

### Outcome Variables

#### Overview

The primary outcome variable was cell phone use, a self-reported binary outcome defined as owning a cell phone and reporting that it was turned on for at least half of each day. Two secondary outcomes were frequent sending and receiving of text messages. Participants were asked 2 separate questions about how frequently they send and receive SMS text messages: several times per day, several times per week, rarely, or never. These were binary outcomes defined as “yes” if the participant self-reported sending and receiving text messages several times a week or more.

We categorized them as above because for mHealth interventions to be effective, participants need to have a working phone that is turned on most of the time and text messaging several times a week for SMS interventions to be useful.

#### Main Independent Variable

Alcohol use was assessed on the basis of self-report and Phosphatidylethanol levels. Participants were administered the Alcohol Use Disorders Identification Test–alcohol consumption questions [20], which was modified to ask about drinking in the prior 3 months. A score of ≥3 was considered indicative of an Alcohol Use Disorders Identification Test – Consumption positive status for women and ≥4 was considered positive for men [21]. For the main analyses, we defined alcohol use as any self-reported alcohol use in the last 3 months or a Phosphatidylethanol level of ≥8 ng/mL [22]. In exploratory analyses, we examined the level of alcohol use as a 3-level variable, defined as follows: “none” implying no self-reported alcohol use in the prior 3 months and a Phosphatidylethanol level of <8 ng/mL, “moderate” implying any self-reported alcohol use in the prior 3 months but an Alcohol Use Disorders Identification Test – Consumption negative status and a Phosphatidylethanol level of ≥8 to <50 ng/mL, and “heavy” implying an Alcohol Use Disorders Identification Test – Consumption positive status or a Phosphatidylethanol level of ≥50 ng/mL.

#### Covariates

We controlled for participant characteristics such as age, sex, literacy, level of education, household asset index, and organized religious activity because of their prior [23-26] or suspected associations with alcohol use and cell phone use. Level of literacy was assessed by asking the participants to read a sentence in the language they are most comfortable with; we considered those who were able to read all or part of the sentence to be literate. The household asset index (HHI) was created using principal component analysis (PCA) [27] and was based on durable goods, housing quality, and available energy sources. Cell phones were excluded from the list of durable goods included in the PCA. We dichotomized this measure as low (bottom 40%) and medium or high (top 60%) because of concern about small cell sizes in multivariable analyses.

We assessed organized religious activity by asking about the frequency of attending religious activities using the Duke University Religion Index [28], and categorized it as weekly or more versus less than weekly attendance of religious activity.

### Statistical Analyses

We described sample characteristics using proportions for categorical variables and median (IQR) values for continuous variables. We used unadjusted and adjusted logistic regression models to examine associations with cell phone use (primary outcome), and sending and receiving text messages (secondary outcomes). The multivariable models controlled for the following potential confounders selected a priori: age, sex, literacy, HHI, and organized religious activity. In the multivariable model for frequent sending of text messages, we included education instead of literacy owing to a zero cell (ie, no one in the nonliterate group reported sending text messages frequently). Finally, we conducted exploratory analyses using the 3-level alcohol use variable rather than any alcohol use as the main independent variable of interest.

## Results

### Results Overview

A total of 1434 people were screened for the ADEPTT study. Main reasons for ineligibility included a history of active TB (n=26) or TB medications (n=42), elevated AST or ALT (n=79), and testing negative on the TST (n=848). Of the screened participants, 308 people were found to be eligible for enrollment. Six people declined enrollment, one was later found to be ineligible and was excluded, and one was missing cell phone use data; 300 participants were included in this analysis.

Of the 300 participants included, approximately half of them (n=146, 48.7%) were male and their median age was 40 (IQR 33-47) years (Table 1). The majority of the participants (n=219, 73.0%) had no more than primary school (6 years) education. Two-thirds (n= 199, 66.6%) of the participants were either married or cohabiting. Approximately half (n=158, 52.7%) of the participants reported attending organized religious services at least once a week. The majority of the participants (n=263, 87.7%) were literate. By design, over two-thirds (n=214, 71.3%) of participants had consumed alcohol in the past 3 months.

**Table 1:** Baseline characteristics of study participants with data on cell phone use in Mbarara, Uganda (N=300).

Variables				Values
Age (years), median (IQR)				40 (33-47)
**Sex, n (%)**				
	Female			154 (51.3)
	Male			146 (48.7)
**Marital status, n (%)**				
	No			100 (33.4)
	Yes			199 (66.6)
**Education, n (%)**				
	Primary or less			219 (73.0)
	More than primary			81 (27.0)
**Literate, n (%)**				
	No			37 (12.3)
	Yes			263 (87.7)
**Employment status, n (%)**				
	Unemployed			12 (4.0)
	Employed			288 (96.0)
**Household asset index, n (%)**				
	Low			120 (40.1)
	Middle or high			179 (59.9)
**Organized religiosity: frequency of attending religious services, n (%)**				
	Less than weekly			142 (47.3)
	Weekly or more			158 (52.7)
**Any alcohol use, prior 3 months^a^, n (%)**				
	No			85 (28.4)
	Yes			214 (71.3)
**Level of alcohol use, prior 3 months^b^, n (%)**				
	None			85 (28.4)
	Moderate			66 (22.1)
	Heavy			148 (49.5)
**Risky sexual behavior^c^, n (%)**				
	No			297 (99.3)
	Yes			2 (0.7)
**Cell phone use, n (%)**				
	**Has access to use a cell phone**			
		No	33 (11.0)	
		Yes	267 (89.0)	
	**Shared a cell phone (n=267 persons with cell phone access)**			
		No	241 (90.3)	
		Yes	26 (9.7)	
	**Owns a cell phone (n=267)**			
		No	5 (1.9)	
		Yes	262 (98.1)	
	**For how much of the time is the phone on (n=267)**			
		Most of the time	259 (97.0)	
		Approximately half of the time	7 (2.6)	
		Less than half of the time	1 (0.4)	
**Cell phone use: summary**				
	Does not own a phone, or phone is on less than half of the time			38 (12.7)
	Owns a phone and it is on half of the time or more			262 (87.3)
**Frequency of text message (SMS) receipt**				
	Rarely or never			200 (66.9)
	Several times a week or more			99 (33.1)
**Frequency of sending text messages (SMS)**				
	Rarely or never			269 (89.7)
	Several times a week or more			31 (10.3)

^a^Any alcohol use in the prior 3 months was defined as any alcohol use self-reported in the prior 3 months or a phosphatidylethanol level of ≥8 ng/mL.

^b^Level of alcohol use in the prior 3 months defined as follows: “none” implies no self-reported alcohol use in the prior 3 months and a Phosphatidylethanol level of <8 ng/mL; “moderate” implies any self-reported alcohol use in the prior 3 months but an Alcohol Use Disorders Identification Test–alcohol consumption questions (AUDIT-C) negative status and a Phosphatidylethanol level of ≥8 to <50 ng/mL; “heavy” implies an AUDIT-C positive status or a Phosphatidylethanol level of ≥50 ng/mL.

^c^Risky sexual behavior implies having had unprotected sex with someone who was not a husband, wife, or steady partner during the participant’s most recent sexual encounter.

### Cell Phone Use

The majority (n=267, 89.0%) of participants reported they had access to a cell phone; of them, 262 (98.1%) owned the phone and 26 (9.7%) shared the phone. The majority (n=262, 87.3%) of participants used a cell phone (owned a cell phone and had it on for more than half of the day; Table 1). In unadjusted analysis, any alcohol use was not significantly associated with cell phone use (odds ratio [OR] 0.76, 95% CI 0.34-1.67; *P*=.49) (Table 2). Adjusted analyses showed lower odds of cell phone use among those who consumed alcohol than among those who did not, although this did not reach statistical significance (adjusted OR [aOR] 0.48, 95% CI 0.18-1.25; *P*=.13).

We observed associations between literacy and cell phone use (aOR 4.15, 95% CI 1.74-9.89; *P*<.01) and also between middle or high HHI and cell phone use (aOR 8.03, 95% CI 3.26-19.79; *P*<.01).

**Table 2:** Unadjusted and adjusted associations with cell phone use.

Variable		Participants who do not own a phone or whose phone is on for less than half the time (n=38)	Participants who own a phone that is on for greater than or equal to half of the time (n=262)	Unadjusted odds ratio (95% CI)	*P* value		Adjusted odds ratio^a^ (95% CI)		*P* value	
**Any alcohol use, prior 3 months^b^, n (%)**						0.49				.13
	No	9 (10.6)	76 (89.4)	1.00 (reference)			1.00 (reference)			
	Yes	29 (13.6)	185 (86.5)	0.76 (0.34-1.67)			0.48 (0.18-1.25)			
Age (years), median (IQR)		42 (35-47)	40 (33-47)	0.99 (0.95-1.02)	.46		1.00 (0.96-1.04)		>.99	
**Sex, n (%)**						.39				.28
	Female	22 (14.3)	132 (85.7)	1.00 (reference)			1.00 (reference)			
	Male	16 (11.0)	130 (89.0)	1.35 (0.68-2.69)			1.61 (0.68-3.78)			
**Literate, n (%)**						<.001				.001
	No	14 (37.8)	23 (62.2)	1.00 (reference)			1.00 (reference)			
	Yes	24 (9.1)	239 (90.9)	6.06 (2.76-13.30)			4.15 (1.74-9.89)			
**Household asset index, n (%)**						<.001				<.001
	Low	31 (25.8)	89 (74.2)	1.00 (reference)			1.00 (reference)			
	Middle or high	7 (3.9)	172 (96.1)	8.56 (3.62-20.21)			8.03 (3.26-19.79)			
**Organized religiosity: frequency of attending religious services, n (%)**						.73				.79
	Less than weekly	19 (13.4)	123 (86.6)	1.00 (reference)			1.00 (reference)			
	Weekly or more	19 (12.0)	139 (88.0)	1.13 (0.57-2.23)			1.11 (0.50-2.46)			

^a^Calculated with 298 participants.

^b^Any alcohol use in the prior 3 months was defined as any alcohol use self-reported in the prior 3 months or a phosphatidylethanol level of ≥8 ng/mL.

### Exploratory Analysis

We performed exploratory analyses to assess the impact of level of drinking on cell phone use but did not find an association between level of alcohol use (moderate and heavy vs no use) and cell phone use (moderate use: aOR 0.62, 95% CI 0.19-2.04; heavy use: aOR 0.42, 95% CI 0.15-1.17; *P*=.25; Table 3).

**Table 3:** Adjusted odds ratios (aORs) and 95% CIs for cell phone use.

Parameters		aOR (95% CI)	*P* value	
**Level of alcohol use, prior 3 months^a^**				.25
	None	1.00 (reference)		
	Moderate	0.62 (0.19-2.04)		
	Heavy	0.42 (0.15-1.17)		
Age (per 1 year)		1.00 (0.96-1.04)	.94	
**Sex**				.21
	Female	1.00 (reference)		
	Male	1.78 (0.72-4.38)		
**Literate**				.001
	No	1.00 (reference)		
	Yes	4.08 (1.71-9.69)		
**Household asset index**				<.001
	Low	1.00 (reference)		
	Middle/High	7.98 (3.23-19.70)		
**Organized religiosity: frequency of attending religious services**				.76
	Less than weekly	1.00 (reference)		
	Weekly or more	1.13 (0.51-2.50)		

^a^Level of alcohol use in the prior 3 months defined as follows: “none” implies no self-reported alcohol use in the prior 3 months and a phosphatidylethanol level of <8 ng/mL; “moderate” implies any self-reported alcohol use in the prior 3 months but an Alcohol Use Disorders Identification Test–alcohol consumption questions (AUDIT-C) negative status and a Phosphatidylethanol level of ≥8 to <50 ng/mL; and “heavy” implies an AUDIT-C positive status or a Phosphatidylethanol level of ≥50 ng/mL.

### Text Message (SMS) Use

Over two-thirds (n=200, 66.9%) of the total participants reported rarely or never receiving text messages and the majority (n=269, 89.7%) reported rarely or never sending text messages (Table 1). Of those who had access to a cell phone (n=267), only one-third (99/267, 37.1%) reported frequently receiving text messages, while only (31/267, 11.6%) reported frequently sending text messages.

In adjusted analyses, any alcohol use was not significantly associated with frequent sending (aOR 0.82, 95% CI 0.28-2.37; *P*=.71) or receiving (aOR 1.31, 95% CI 0.70-2.47; *P*=.40) text messages (Tables 4 and 5).

Age was significantly associated with frequent sending of text messages (aOR 0.93, 95% CI 0.88-0.98; *P*<.01) but not with frequent receiving of text messages (aOR 0.98, 95% CI 0.95-1.00; *P*=.09) (Tables 4 and 5).

**Table 4:** Unadjusted and adjusted associations with receiving text messages (SMS) several times a week or more.

Variables			Frequency of receiving text messages					Unadjusted odds ratio (95% CI)			*P* value				Adjusted odds ratio^a^ (95% CI)				*P* value	
			Rarely or never (n=200)		Several times a week or more (n=99)															
**Any alcohol use, prior 3 months^b^, n (%)**												.21								.40
	No	61 (72.6)		23 (27.4)		1.00 (reference)							1.00 (reference)							
	Yes	139 (65.0)		75 (35.1)		1.43 (0.82-2.49)							1.31 (0.70-2.47)							
Age (years), median (IQR)			40 (35-48)		37 (31-45)		0.98 (0.95-1.00)			.06				0.98 (0.95-1.00)				.09		
**Sex, n (%)**												.51								.47
	Female	105 (68.6)		48 (31.4)		1.00 (reference)							1.00 (reference)							
	Male	95 (65.1)		51 (34.9)		1.17 (0.73-1.90)							1.24 (0.69-2.22)							
**Education, n (%)**												<.001								
	Primary or less	164 (75.2)		54 (24.8)		1.00 (reference)							—^c^							
	More than primary	36 (44.4)		45 (55.6)		3.80 (2.22-6.48)							—							
**Literate, n (%)**												.006								.03
	No	32 (88.9)		4 (11.1)		1.00 (reference)							1.00 (reference)							
	Yes	168 (63.9)		95 (36.1)		4.52 (1.55-13.18)							3.41 (1.13-10.36)							
**Household asset index, n (%)**												<.001								.001
	Low	96 (80.7)		23 (19.3)		1.00 (reference)							1.00 (reference)							
	Middle or high	103 (57.5)		76 (42.5)		3.08 (1.79-5.30)							2.62 (1.50-4.58)							
**Organized religiosity: frequency of attending religious services, n (%)**												.62								.33
	Less than weekly	97 (68.3)		45 (31.7)		1.00 (reference)							1.00 (reference)							
	Weekly or more	103 (65.6)		54 (34.4)		1.13 (0.70-1.83)							1.31 (0.76-2.26)							

^a^Calculated with 297 participants.

^b^Any alcohol use in the prior 3 months was defined as any alcohol use self-reported in the prior 3 months or a phosphatidylethanol level of ≥8 ng/mL.

^c^Not determined.

**Table 5:** Unadjusted and adjusted associations with frequency of sending text messages (SMS) several times a week or more.

Variable		Frequency of sending text messages		Unadjusted odds ratio (95% CI)	*P* value		Adjusted odds ratio^a^ (95% CI)		*P* value	
		Rarely or never (n=269)	Several times a week or more (n=31)							
**Any alcohol use, prior 3 months^b^, n (%)**						.73				.71
	No	77 (90.6)	8 (9.4)	1.00 (reference)			1.00 (reference)			
	Yes	191 (89.3)	23 (10.8)	1.16 (0.50-2.70)			0.82 (0.28-2.37)			
Age (years), median (IQR)		40 (34-48)	34 (29-40)	0.93 (0.89-0.98)	<.001		0.93 (0.88-0.98)		.005	
**Sex, n (%)**						.68				.46
	Female	137 (89.0)	17 (11.0)	1.00 (reference)			1.00 (reference)			
	Male	132 (90.4)	14 (9.6)	0.85 (0.41-1.80)			0.69 (0.26-1.83)			
**Education, n (%)**						<.001				<.001
	Primary or less	211 (96.4)	8 (3.7)	1.00 (reference)			1.00 (reference)			
	More than primary	58 (71.6)	23 (28.4)	10.46 (4.45-24.60)			10.35 (4.02-26.60)			
**Literate, n (%)**										
	No	37 (100)	0 (0.0)	—^c^			—			
	Yes	232 (88.2)	31 (11.8)	—			—			
**Household asset index, n (%)**						0.007				.40
	Low	115 (95.8)	5 (4.2)	1.00 (reference)			1.00 (reference)			
	Middle/High	153 (85.5)	26 (14.5)	3.91 (1.46-10.49)			1.60 (0.53-4.80)			
**Organized religiosity: frequency of attending religious services**						.05				.08
	Less than weekly	122 (85.9)	20 (14.1)	1.00 (reference)			1.00 (reference)			
	Weekly or more	147 (93.0)	11 (7.0)	0.46 (0.21-0.99)			0.43 (0.17-1.09)			

^a^Calculated with 298 participants.

^b^Any alcohol use in the prior 3 months was defined as any alcohol use self-reported in the prior 3 months or a phosphatidylethanol level of ≥8 ng/mL.

^c^Not determined.

### Exploratory Analysis

We performed exploratory analyses to assess the impact of level of drinking on frequent sending and receiving of text messages and found an association between level of alcohol use and frequent receipt of text messages (aORs for moderate and heavy alcohol use, respectively: 0.76, 95% CI 0.35-1.65 and 1.84, 95% CI 0.93-3.66; *P*=.03), but not with frequent sending of text messages (aORs for moderate and heavy alcohol use, respectively: 0.64, 95% CI 0.17-2.31 and 0.96, 95% CI 0.30-3.05; *P*=.73; Table 6).

**Table 6:** Adjusted odds ratios (aORs) and 95% CIs for sending and receiving text messages (SMS) frequently.

Variables			Frequent receipt of text messages (n=297)							Frequent sending of text messages (n=298)							
			aOR (95% CI)		*P* value			aOR (95% CI)				*P* value					
**Level of alcohol use, prior 3 months^a^**						.03							.73				
	None	1.00 (reference)					1.00 (reference)										
	Moderate	0.76 (0.35-1.65)					0.64 (0.17-2.31)										
	Heavy	1.84 (0.93-3.66)					0.96 (0.30-3.05)										
Age (per 1 year)			0.98 (0.95-1.01)		.12			0.93 (0.88-0.98)				.007					
**Sex**						.91							.37				
	Female	1.00 (reference)					1.00 (reference)										
	Male	1.04 (0.56-1.90)					0.63 (0.23-1.73)										
**Literate**						.02											
	No	1.00 (reference)					—^b^										
	Yes	4.04 (1.30-12.57)					—										
**Education**													<.001				
	Primary or less	—					1.00 (reference)										
	More than primary	—					10.28 (3.99-26.51)										
**Household asset index**						<.001							.37				
	Low	1.00 (reference)					1.00 (reference)										
	Middle or high	2.85 (1.62-5.03)					1.65 (0.55-4.99)										
**Organized religiosity: frequency of attending religious services**						.34							.07				
	Less than weekly	1.00 (reference)					1.00 (reference)										
	Weekly or more	1.31 (0.75-2.27)					0.43 (0.17-1.08)										

^a^Level of alcohol use in the prior 3 months defined as follows: “none” implies no self-reported alcohol use in the prior 3 months and a phosphatidylethanol level of <8 ng/mL; “moderate” implies any self-reported alcohol use in the prior 3 months but an Alcohol Use Disorders Identification Test–alcohol consumption questions (AUDIT-C) negative status and a Phosphatidylethanol level of ≥8 to <50 ng/mL; “heavy” implies a AUDIT-C positive status or a Phosphatidylethanol level of ≥50 ng/mL.

^b^Not determined.

## Discussion

### Principal Findings

We evaluated cell phone ownership and use (frequency of sending and receiving text messages) in a cohort of persons living with HIV in HIV care, who were coinfected with latent TB in Uganda. We also evaluated the association between alcohol use and cell phone use. The results showed a high prevalence of both cell phone ownership and use in this population. This is consistent with many other studies and surveys of cell phone access and use in Uganda [3,29] and in Africa [30]. This high prevalence of cell phone ownership may be largely attributed to the ease of use and relatively low cost of cell phones [11,31]. Persons living with HIV have been shown to find cell phones convenient as an mHealth tool [32] and have reported that it helps reduce transportation costs and still provides access to much needed health information [3]. We found high levels of cell phone use, indicating the potential utility of cell phone ownership in providing care and treatment for persons living with HIV, and as a tool that can be leveraged for mHealth and interventions.

In this analysis, alcohol use was not significantly associated with cell phone use. This finding held up in exploratory analyses examining the level of drinking. Our findings were different from a study conducted in Uganda among persons living with HIV in a fishing community that found that cell phone use was associated with alcohol use before sexual intercourse, although this was only among young females aged 14-24 years [26]. Other studies, conducted in high-resource settings, in the general population (ie, not focused on persons living with HIV) and among young persons, have found positive associations between alcohol use and cell phone use [33,34]. The differences observed may be due to the fact that in these other studies, the population was much younger than that in our study. In our study, the median age was 40 years while many of these studies were among younger persons. Younger persons may use cell phones for social reasons, including making plans for drinking and entertainment [35] with peers, while older persons may use cell phones more for economic reasons, as they have to provide for families and have more responsibilities; hence, we observed a lack of association with alcohol and instead observed an association with HHI and literacy. These findings suggest the need to carefully evaluate the target population for cell phone–based interventions, and that these interventions should take into consideration the level of literacy and economic status of the population.

We also found that participants rarely sent or received text messages (approximately 30%). This was different from other studies, especially those conducted in middle- and high-resource countries where sending and receiving of text messages was high (60%) [36], but similar to a study among youths with HIV in Uganda (27%) [5]. A suggested explanation for the low text message use is the loss of novelty of sending or receiving text messages on a mobile phone in Africa [5] and therefore decreased use of the function. A great majority of the participants were literate; literacy was associated with frequent receiving of texts, and none of the persons who frequently sent text messages were nonliterate. While we could not include literacy in the model because no nonliterate person reported sending text messages; there was a strong effect of education with sending of text messages, which is consistent with prior reports [37,38]. We did not observe an association between any alcohol use and frequent sending and receiving of text messages; however, the exploratory analysis suggests that level of alcohol use may be associated with odds of frequent receipt of text messages. While cell phone use was high, using text messages alone as an mHealth intervention may not be effective, especially with interventions that require responses.

### Limitations of the Study

This study was limited in the fact that the sample was selected for the main study enrollment purposes (ie, having latent TB); this may limit the generalizability of the study. However, we did not observe any major differences between patients enrolled and those not enrolled when we analyzed major factors associated with one of the selection criteria (PPD+ skin test) [39]. Another limitation is that this study may have been underpowered to detect associations, as it was not designed to evaluate the relationships being examined. In a post hoc power calculation, we found the study would have approximately 80% power to detect an odds ratio of 0.34 or lower for the relationship between any alcohol use and any cell phone use, assuming 89% cell phone use among those without alcohol use as observed in our sample. The study was, therefore, likely underpowered to detect the magnitude of the association observed in this cohort.

### Strengths of the Study

The strength of this study was that alcohol use was measured both by self-report and objectively by use of a biomarker to confirm actual alcohol use.

### Conclusions

Many persons living with HIV own cell phones that are turned on for more than half of the day. There is, however, very little use of text messaging among persons living with HIV in this low-income setting.

There is hope that mHealth interventions in this population can be carried out using cell phones owing to their popularity; however, the interventions may need to employ methods that do not rely exclusively on the sending and receiving of text messages. Future mixed methods studies with large sample sizes should be conducted to further evaluate preferred alternatives to text messages such as interactive voice response and the relationship between alcohol use and cell phone use.

## Abbreviations

ADEPTT: Alcohol Drinkers’ Exposure to Preventative Therapy for Tuberculosis
ALT: alanine transaminase
ART: antiretroviral therapy
AST: aspartate transaminase
AUDIT-C: Alcohol Use Disorders Identification Test–alcohol consumption questions
HHI: household asset index
INH: isoniazid
PCA: principal component analysis
PPD: purified protein derivative
TB: tuberculosis
TST: tuberculin skin test
